# Standards of practice in forensic age estimation with CT of the medial clavicular epiphysis—a systematic review

**DOI:** 10.1007/s00414-023-03061-7

**Published:** 2023-09-11

**Authors:** Thomas D. Ruder, Saskia C. Kuhnen, Wolf-Dieter Zech, Jeremias B. Klaus, Paolo Lombardo, Michael Ith

**Affiliations:** 1 Institute of Diagnostic, Interventional and Pediatric Radiology, InselspitaI, Bern University Hospital, University of Bern, Freiburgstrasse, CH-3010 Bern, Switzerland; 2 Institute of Forensic Medicine, University of Bern, Bern, Switzerland; 3 Roentgen Institute Thun, Thun, Switzerland; 4 Radiation Protection, Image Processing Systems & Radiological Processes, Hirslanden Private Hospital Group, Zurich, Switzerland

**Keywords:** Forensic age estimation, Medial clavicular epiphysis, Computed tomography, CT parameters, Systematic review

## Abstract

The AGFAD (Arbeitsgemeinschaft für Forensische Alterdiagnostik, Study Group on Forensic Age Diagnostics) has published several recommendations regarding both technical aspects of computed tomography (CT) of the medial clavicular epiphysis (MCE) and the process of reading and interpreting the CT images for forensic age estimations (FAE). There are, however, no published recommendations regarding CT scan protocols and no dose reference values for CT of the MCE. The objective of this analysis was to assess adherence to AGFAD recommendations among practitioners of FAE and analyse reported dose-relevant CT scan parameters with the objective of helping to establish evidence-based dose reference values for FAE. A systematic literature search was conducted in PubMed and in Google Scholar with specific MeSH terms to identify original research articles on FAE with CT of the MCE from 1997 to 2022. A total of 48 studies were included. Adherence to AGFAD recommendations among practitioners of FAE is high regarding the use of Schmeling main stages (93%), bone window (79%), ≤ 1 mm CT slices (67%), axial/coronal CT images (65%), and Kellinghaus sub-stages (59%). The reporting of CT technique and CT dose-relevant scan parameters is heterogeneous and often incomplete in the current literature. Considering the success achieved by the AGFAD in creating standards of practice of FAE in living subjects, there is potential for the AGFAD to establish standards for radiation protection in FAE as well.

## Introduction

The AGFAD (Arbeitsgemeinschaft für Forensische Alterdiagnostik, Study Group on Forensic Age Diagnostics) provides guidance on forensic age estimation (FAE) of the living: for FAE in young, potentially minor individuals of uncertified age, radiologic evaluation includes x-ray images of the left hand and teeth to estimate bone and dental age, respectively, as well as computed tomography (CT) of the medial clavicular epiphysis (MCE) in individuals who have reached skeletal maturity of the hand [1–5].

The AGFAD recommends using the Schmeling and Kellinghaus classification systems to evaluate the main stage and sub-stage of MCE ossification on CT scans, and advises that two experts should provide age estimates using mutliplanar reformatted (MPR) axial and coronal images with a slice thickness of 1 mm or less and CT window settings optimized for bone viewing [6–16].

The AGFAD also recommends applying the minimum age concept in the assessment of age estimations to ensure that the age of the person being assessed is not overestimated [3, 5]. This practice also addresses the issue of age mimicry highlighted by Ding [17].

A recent systematic review by Buckley has found that CT currently represents the preferred imaging modality for FAE for many practitioners [18]: CT is more accessible and cost-effective compared to MRI and exhibits lower reader-dependency compared to x-ray and ultrasound. Additionally, CT provides a substantial pool of international reference data on the evolution of MCE [6, 18]. The primary limitation of CT imaging is its use of ionizing radiation.

Therefore, radiation protection is a crucial aspect of CT imaging, particularly in the context of FAE where CT is performed for non-medical reasons on a young population, and limiting the scan length to the recommended 4 cm [3, 9, 11, 20–22]) is one way to reduce radiation exposure in FAE [9, 16, 23, 24]. According to the literature, the effective dose values for CT of the MCE, as estimated and calculated, tend to be low, typically below 1 millisievert (mSv), but can vary from 0.2 to 4.6 mSv depending on the scan protocol [11, 15, 16, 21, 22, 24]. There are currently no established dose reference values and no generally accepted scan settings for CT of the MCE for FAE [15, 16].

The objective of this systematic literature review is to (1) assess adherence to AGAFD recommendations among practitioners of FAE and (2) analyse reported dose-relevant CT scan parameters of CT of the MCE for FAE with the objective of helping to establish evidence-based dose reference values for CT of the MCE for FAE.

## Method

### Literature search

A systematic literature search was conducted in MEDLINE (PubMed) and GoogleScholar. The aim was to identify original research articles on age estimation with CT of the medical clavicular epiphysis. For this purpose, a search equation with the following Medical Subject Headings (MeSH) terms was created for PubMed: ((age estimation OR age determination) AND (clavicular OR clavicle OR medial clavicular epiphysis OR sternoclavicular joints) AND (CT OR CT scan OR computed tomography OR scanner NOT MRI OR magnetic resonance imaging)), filtered by publication date 1997/01/01–2022/11/01. In addition, Pubmed citation searches were conducted for publications citing the following four FAE landmark articles by Kreitner [20], Schmeling [1], and Kellinghaus [8, 27].

Two independent reviewers performed the literature search. The reference lists of all articles assessed for eligibility were searched for additional publications.

### Data extraction and analysis

For each of the included studies, the following six categories of information were extracted:

1. Publication information (authors and affiliations, date of (online) publication, journal name, and category, and geographic origin)
2. Indication of CT scans (clinical query, post-mortem investigation, age estimation)
3. Reported CT technique (scanned body region, scan length, position of arms during scan).
4. Reported CT scan parameters (kilovolt (kVp), milliampere-seconds (mAs), use of automatic tube current modulation).
5. Reported radiation dose information (CT dose index (CTDI), dose length product (DLP), and effective dose).
6. Adherence to AGFAD recommendations (use of Schmeling main stages, use Kellinghaus sub-stages, use ≤ 1 mm CT slices, use of bone window, use of axial/coronal multiplanar reformats (MPR)).

All parameters were exported for quantitative analysis. Categorical variables and qualitative parameters are expressed as frequencies and percentage (%). Excel (Microsoft Excel, 2010 Microsoft Corporation, Redmond, WA, USA) was used to calculate descriptive statistics.

## Results

### Literature search

**Fig. 1:**
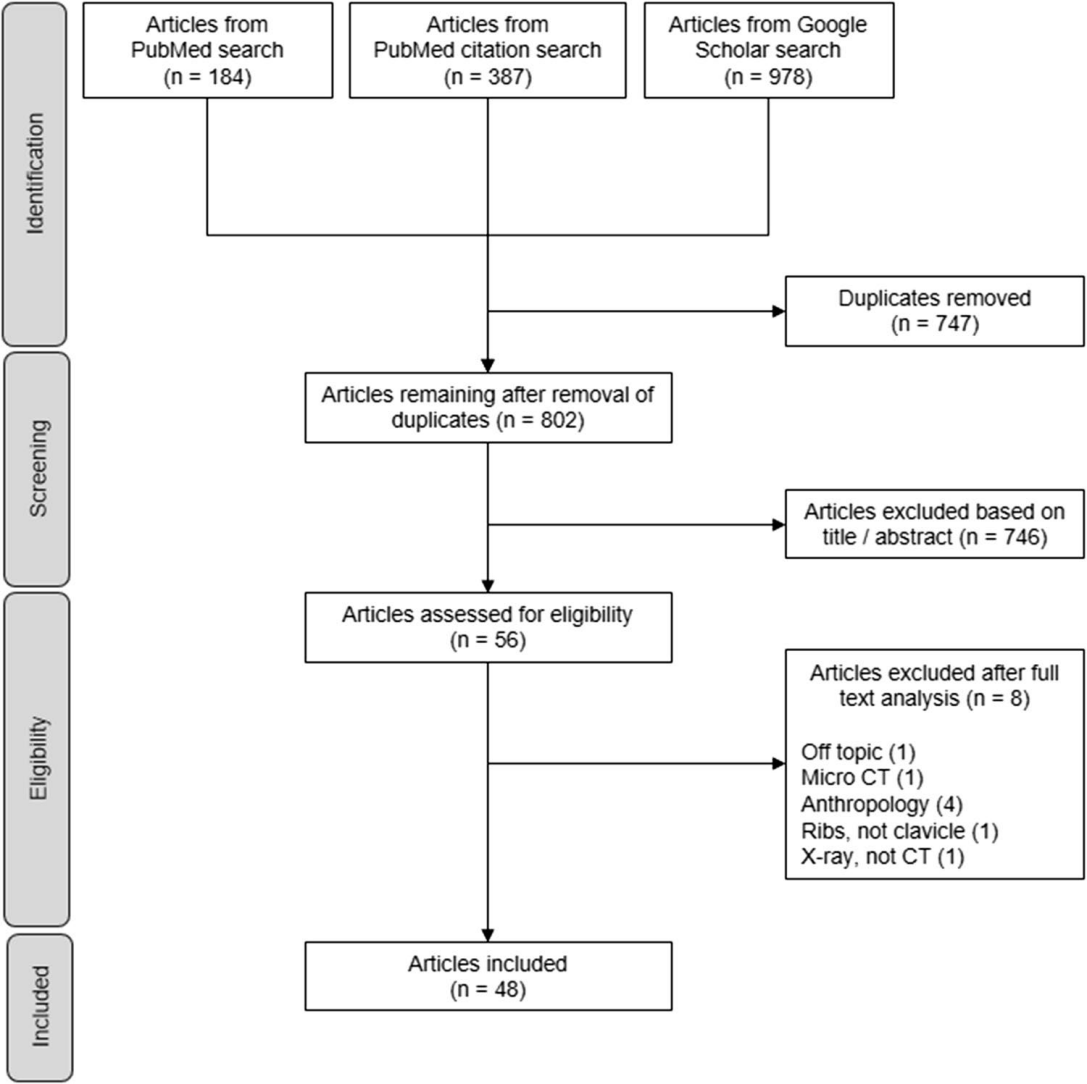
Flow diagram of literature search and selection process

The process of literature identification, screening, eligibility, and inclusion is visualized in the flow diagram below (Fig. 1). The PubMed literature search and the citation search identified 184 and 387 articles, respectively (*n* = 571), with 62 citations for Kreitner’s article [20], 169 for Schmeling’s article [1], and 79 and 77 citations for the two Kellinghaus articles [8, 27]. The GoogleScholar search identified 978 articles (*n* = 978). After elimination of duplicates, the remaining 802 articles were screened and 56 were included for full-text analysis. Of these, 48 studies were included in the systematic review [7, 8, 10–16, 19, 20, 24–60].

### Publication information

Only 7 of all 48 studies (15%) were published during the first half of the study period from 1997 to 2009. Most articles (41/48, 85%) were published between 2010 and 2022. In 32/48 (67%) publications both forensic pathologists and radiologists are involved as co-authors. In 12/48 (25%) and 4/48 (8%) publications there is no affiliation with a radiologic institute or forensic institute, respectively. An overwhelming majority of articles (42/48, 88%) were published in forensic sciences/legal medicine journals. 4/48 articles were published in radiologic journals (8%), 1/48 in a forensic imaging journal (2%). Most publications originated from Europe (26/48, 54%), followed by Asia (16/48, 33%), Australia (4/48, 8%), and Africa (2/48, 4%) (Table 1).

**Table 1:** Overview of publication information

Continent	*n*	Country	*n*	Affiliation FM & RX	Affiliation FM	Affiliation RX
Europe	26	Germany	17	[7], [8], [11], [12], [13], [14], [15], [16], [19]*, [20]*, [25], [27], [28], [41]	[34]	[10], [24]*
		Austria	4	[53], [56], [57]		[40]
		France	3	[60]	[46], [47]	
		Denmark	1	[33]^#^		
		Serbia	1	[37]		
Asia	16	Turkey	6	[39], [44], [48], [51], [52]		[49]*
		India	4	[29], [54], [58]	[41]	
		China	2		[38], [43]	
		Japan	1		[55]	
		Sri Lanka	2	[59]	[50]	
		Thailand	1	[26]		
Australia	4	Australia	4		[30], [31], [32], [41]	
Africa	2	Egypt	2	[35], [44]		

*n* number of publications for each category, *FM & RX* publications with author affiliations to both institutes of forensic medicine and radiology, *FP* publications with author affiliations to institutes of forensic medicine pathology (but not radiology), *RX* publications with author affiliations to institutes of radiology (but not forensic medicine), *published in radiology journals, ^#^published in forensic radiology journal. Articles without * or ^#^ were published in forensic sciences/legal medicine journals

### Indication of CT scans

The indications of CT scans fall into three main categories: clinical query, post-mortem investigation, and age estimation. Clinical CT scans of individuals with known age (26/48, 54%) as well as post-mortem CT (PMCT) scans of individuals with known age (11/48, 23%) were used to establish reference values for age estimation. 11/48 studies (23%) analyzed CT data of individuals that had been scanned for the purpose of FAE in the living (Table 2).

**Table 2:** Overview of CT indication and scanned body region

Origin of CT data	*n*	Age	*n*	Body region	References
Clinical query	26	Known	9	Chest	[24], [37], [42], [46], [48], [49], [51], [52], [58]
			12	Multiple body regions	[10], [19], [20], [26], [29], [36], [38], [39], [44], [47], [54], [59]
			4	Multiple trauma	[7], [8], [27], [35]
			1	SCJ	[43]
Post mortem investigation	11	Known	6	SCJ	[12], [13], [14], [25], [34], [41]
			5	Whole body	[30], [31], [32], [33], [55]
Forensic age estimation	11	Unknown	10	SCJ	[11], [15], [16], [28], [40], [50], [53], [56], [57], [60]
		Known	1	SCJ	[45]

*n* refers to number of studies per category, *Age* age of individuals in whom age estimation was performed, *SCJ* sternoclavicular joint

### Reported CT technique

The scanned body region was limited to the sternoclavicular joints (SCJ) in 18/48 studies (38%). Among these were all 11 studies of living individuals scanned for FAE, 6 post-mortem studies with PMCTs of excised sternoclavicular joints, and 1 study with clinical CT data, where only “patients undergoing a CT scan of the clavicle” were included. The remaining 25/26 studies with clinical CT data included: chest CT (9/25), multiple trauma CT (4/25), multiple body regions (12/25). Finally, 5/11 post-mortem studies used whole-body PMCT data (Table 2).

Arm position during the CT and scan length were reported in 2/48 (one arms-down, one arms-down vs. arms-up) and 5/48 studies respectively (range: 4–8.5 cm) (Table 4).

### Reported CT scan parameter and radiation dose

Reporting of CT scan parameters was heterogeneous: 36/48 (75%) reported kVp, 30/48 (63%) reported mAs (including reporting of mA, reference mAs, and effective mAs), and 15/48 (31%) reported use of automatic tube current modulation. Information regarding CT dose was scarce. Only 5/48 studies report dose parameters [11, 15, 16, 24, 29]. Of these, 3 studies report complete dose information (CTDI, DLP, and effective dose [mSv]) [15, 16, 24], while 2 studies report partial dose information (CTDI and effective dose, respectively) [11, 29]. 12/48 provided no information regarding the CT scan parameters and radiation dose. See Table 3 for an overview of results related to CT scan and dose parameters. See Table 4 for a sub-analysis of the 11 studies with published data on FAE of the living.

**Table 3:**
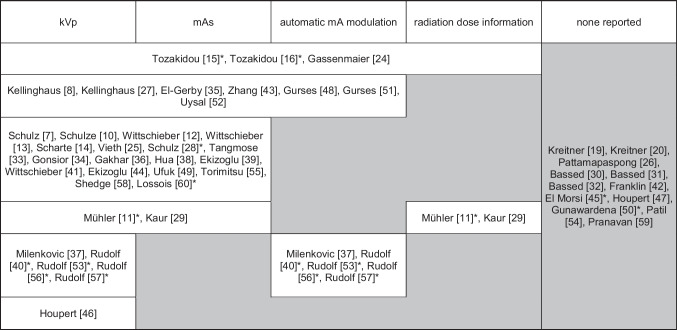
Reported CT scan parameters and dose parameters

*kVp* kilovolt peak (tube potential); *mAs* milliampere-second (tube current - time product); *mA* milliampere (tube current); * studies on age estimation in the living. Reading example: Studies [15] and [16] reported kVp, mAs, use of DMS, and Dose parameters, while [11] reported kVp, mAs, Dose parameters, but not if DMS was used

**Table 4:** Reported CT scan parameters and dose parameters of FAE studies

Author	Published (online)	CT scanner	Arm position	Scan lenght [cm]	kVp	mA/mAs	Automatic mA modulation	Effective dose [mSv]	DLP [mGy*cm]
Mühler [11]	2005	1-slice	Not rep	4	120	130 mA	Not rep	0.5*	Not rep
Schulz [28]	2007	1-slice	Not rep	4	120	130 mA	Not rep	Not rep	Not rep
El Morsi [45]	2015	“Multi-slice CT”	Not rep	Not rep	np	Not rep	Not rep	Not rep	Not rep
Gunawardena [50]	2016	64-slice	Not rep	Not rep	np	Not rep	Not rep	Not rep	Not rep
Rudolf [40]	2014	16-slice	Not rep	Not rep	130	Not rep	Used	Not rep	Not rep
Rudolf [53]	2017	16-slice	Not rep	Not rep	130	Not rep	Used	Not rep	Not rep
Rudolf [56]	2019	16-slice	Not rep	Not rep	130	Not rep	Used	Not rep	Not rep
Rudolf [57]	2019	16-slice	Not rep	Not rep	130	Not rep	Used	Not rep	Not rep
Tozakidou [15]	2019	256-slice	Down	6.2 ± 2.1	140	70 mAs ref	Used	0.95 ± 0.38	69.5 ± 27.6
Tozakidou [16]	2021	256-slice	Down	6.2 ± 2.1	140	70 mAs ref	Used	0.95 ± 0.38	69.5 ± 27.6
			Up	8.5 ± 3.4	140	70 mAs ref	Used	0.79 ± 0.32	57.5 ± 23.4
Lossois [60]	2022	64-slice	Not rep	4	120	200 mAs eff	Not rep	Not rep	Not rep

*kVp* kilovolt peak (tube potential), *mA* milliampere (tube current), *mAs* milliampere-second (tube current–time product), *calculated dose for a patient of 70 kg

### Adherence to AGFAD recommendations

The Schmeling and Kellinghaus classification systems were used in 42/48 (88%) and 22/18, (46%) of all studies, respectively. This translates to 93% of the 45 studies published after Schmeling’s article in 2004 and 59% of the 37 studies published after Kellinghaus’ article in 2010.

Adherence to recommendations regarding the use of ≤ 1 mm CT slices and axial/coronal reformats has grown in parallel to the evolution of CT technology. Overall, ≤ 1 mm CT were used 32/48 (67%) of studies (of which 28 were published in the last decade) while axial/coronal reformats were used in 31/48, (65%) of studies (26 in the last decade). The use of bone window settings to review CT images of the MCE was documented in 38/48 publications (79%). 10/48 publications [12, 31, 38, 40, 46, 48, 51, 52, 58, 60] provide no information regarding CT image windowing (note that some authors may have viewed images using MPR and bone window, but did not report it in the study) (Table 5).

**Table 5:**
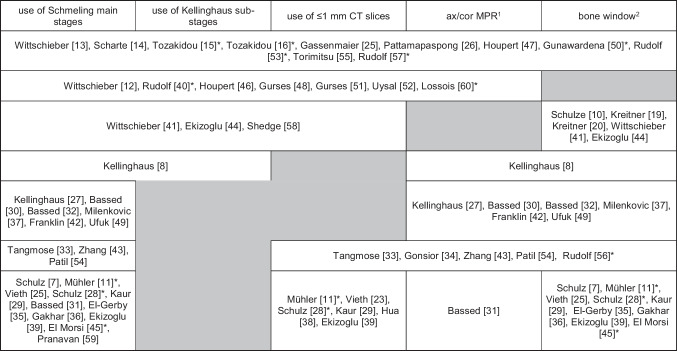
Adherence to AGFAD recommendations

^1^use axial and coronal MPR images reported in study; ^2^ use of bone window in study; * studies on age estimation in the living. Note: some authors may have viewed images using MPR and bone window, but did not report it in the study. Reading example: [41] used both Schmeling main and Kellinghaus sub-stages, and viewed CT images of ≤ 1 mm thickness in a bone window, but they did not report the use of axial and coronal MPR image. [10] used bone window for viewing images

## Discussion

This systematic review on standards of practice in forensic age estimation with CT of the medial clavicular epiphysis has two principal findings: first, there is a considerable level of international standardization in the practice of FAE with CT of the MCE due to high adherence to AGFAD recommendations, and second, reporting of CT technique and CT dose-relevant scan parameters is heterogeneous and often incomplete in the current literature.

These results are relevant because they show how the AGFAD could contribute to the creation of new practice standards regarding dose optimisation for CT of the MCE.

A key finding of this analysis is that reporting of CT technique and scan parameters is incomplete in many studies on CT of clavicles for FAE. The primary reason for this is that more than half of the publications on this topic are reference studies that rely on clinical CT scans of mixed body regions, mixed scan lengths, and therefore mixed CT settings [7, 8, 10, 19, 20, 24, 26, 27, 29, 35–39, 42, 44, 46–49, 51, 52, 54, 58, 59]. Extracting radiation exposure parameters that are meaningful to dedicated CT examinations of the MCE from these data is very challenging. CT scan parameters from post-mortem reference studies are also difficult to transfer to FAE in the living. Although PMCT protocols do not use unreasonable high radiation doses, radiation protection is not a concern in PMCT.

Therefore, the most relevant source of information for guidance on CT technique and scan parameters is those 11 studies that used CT data from CTs of the MCE for actual FAE.

Nine of these do report dose-relevant parameters in their material and methods (two do not) and the specified CT parameters are a good starting point for future work in this field. It is, however, important to keep in mind that of these nine studies, two [11, 28] are more than 15 years old, which is a long time in CT technology. Of the remaining seven studies, four were authored by Rudolf et al. [40, 53, 56, 57], two by Tozakidou et al. [15, 16], and one by Lossois et al. [60]. This means that the available, up-to-date data on CT scan parameters of the MCE for FAE is based on the work of 3 authors only, of which only 1 author had a focus on dose optimisation [15, 16]. These data are insufficient for proposing evidence-based dose reference values for CT of the MCE for FAE.

One important parameter in dose optimisation is arm positioning during CT. According to Tozakidou et al., it is common practice to perform CT of the MCE for FAE in an arms-down position [16]. This systematic review was unable to confirm or deny this practice, because only 2 of all included studies had explicitly reported arm position during CT [15, 16]. The presence of arms within the scan range increases patient dose (more dose required to penetrate humerus) and decreases image quality (more noise from beam hardening artifacts). Clinical practice is therefore to position patient arms outside of the scan range whenever possible, e.g., arms up for chest CT and arms down for neck/c-spine CTs [61–64].

The practice of CT of the MCE with arms down may represent a holdover from the time when age estimations were performed with x-rays of the clavicle (taken in an arms-down position) and should be revaluated in the current era of CT. In this context, one must keep in mind that many CT reference studies for FAE are based on clinical CT scans, often the chest [24, 36, 37, 42, 46, 48, 49, 51, 52, 58]—scanned with arms raised—and therefore, a change in practice to CT of the clavicle with arms-up for age estimation is unlikely to have an effect on staging of MCE ossification. Recently, Tozakidou has already demonstrated how CT of the MCE for FAE with arms up has the potential to decrease effective dose and increases image quality [16].

Finally, another key finding of this analysis is that adherence to AGFAD recommendations is high among practitioners of FAE. Today, the following 5 AGFAD recommendations for age estimation with CT of the clavicles have become internationally recognized standards of practice: (1) use Schmeling main stages (93% adherence after 2004); (2) use of Kellinghaus sub-stages (59% adherence after 2010); (3) use ≤ 1 mm CT images (88% adherence after 2011); (4) use of axial/coronal reformats (80% adherence since 2013); and (5) use of the bone window (documented in 79% of studies).

The fact that the AGFAD has established internationally recognized standards of practice regarding both technical aspects of CT and the process of carrying out the forensic age estimations underlines the potential the AGFAD has to also introduce standards in radiation protection. The fact that the vast majority of publications on FAE with CT of MCE are collaborations between authors with a forensic and authors with a radiologic background is also beneficial to achieve this aim.

Over the past few years, three publications have demonstrated how clinically established dose reduction strategies such as iterative CT reconstruction, arm position outside the scan field, and low dose CT can be transferred to FAE [15, 16, 21].

A recent cadaver study has tested how low CT dose may be lowered while maintaining diagnostic image quality for age estimation [65]. The authors have managed to reduce the dose to approximately 0.15 mSv (calculation with conversion factor of 0.0137 [16]) (using both a fixed parameters protocol at 100 kVp and 30 mAs and a protocol with 120 kVp and automatic dose modulation with 40 mAs reference. These preliminary results indicate that the dose of CT of the MCE may be substantially lowered while still maintaining diagnostic image quality.

## Conclusions

Over the past 2 decades, the AGFAD has published several recommendations regarding both technical aspects of CT and the process of carrying out forensic age estimations that have become internationally accepted standards of practice.

The reporting of dose-relevant CT techniques and CT scan parameters in the literature on FAE is heterogeneous and often incomplete regarding data on dose-relevant CT parameters. Based on the available information, it is not possible yet to propose evidence-based reference values for CT of the MCE for FAE.

In view of the success achieved by the AGFAD in standardizing the practice of FAE in the living, the AGFAD has to potential to establish standards in radiation protection for FAE as well, for example through publishing best practice recommendations for radiation dose optimization.
